# *Paenibacillus* encodes a membrane-localized Spo0B

**DOI:** 10.1128/jb.00367-25

**Published:** 2025-12-29

**Authors:** Isabella N. Lin, Cassidy R. Prince, Heather A. Feaga

**Affiliations:** 1 Department of Microbiology, Cornell University 5922, Ithaca, New York, USA; National Institutes of Health, Bethesda, Maryland, USA

**Keywords:** sporulation, *Paenibacillus*, Spo0B, phosphorelay

## Abstract

Sporulation is a strategy employed by many bacteria to survive harsh environmental conditions. The genus *Paenibacillus* includes spore-forming species notorious for spoiling pasteurized dairy products and for causing American foulbrood in honeybee larvae, leading to colony collapse. Human pathogens within *Paenibacillus* are also a growing threat, causing fatal opportunistic infections. Here, we present a comprehensive survey of sporulation genes across 1,460 high-quality *Paenibacillus* genomes. We find that all members of the sporulation-initiating phosphorelay are well conserved, but that the Spo0B phosphotransferase contains a predicted transmembrane domain. We confirm that this domain localizes Spo0B to the cell membrane and therefore refer to this Spo0B variant as Spo0B-TM. Spo0B-TM is present in 92% of surveyed *Paenibacillus* genomes. Consistent with its high level of conservation, we find that the transmembrane domain is important for detecting its interaction with its phosphorelay partners Spo0A and Spo0F. Moreover, we find that Spo0B exhibits low sequence identity across Bacillota when compared with other members of the phosphorelay. Altogether, this work highlights the potential for diversity even within the highly conserved phosphorelay that initiates sporulation in Bacillota.

**IMPORTANCE:**

The spore is the most durable life form, and the sporulation process serves as a paradigm of cellular development and differentiation. Sporulation is well characterized in the model organism *Bacillus subtilis*, but we lack information about non-model spore formers. The genus *Paenibacillus* includes spore formers that negatively impact farming and food industries and public health. Here, we present the largest comprehensive search for sporulation genes in *Paenibacillus* and show that a unique membrane-localized variant of Spo0B is widespread throughout Paenibacillaceae and is present in other closely related families of Bacilli.

## INTRODUCTION

Endospore formation is a complex developmental process limited to certain classes of Bacillota (1, 2). Through sporulation, vegetative bacteria undergo developmental and metabolic changes to produce highly resistant, dormant spores that are capable of withstanding adverse environmental conditions (3). Spore formation involves a series of morphological stages that are typically triggered by low nutrient density. A vegetative cell asymmetrically divides into a mother cell and a forespore, after which the forespore is fully engulfed and released into the mother cell cytoplasm (4–7). The forespore is encased in two protective shells, the cortex and coat (8–10), and the mature spore is released via lysis of the mother cell (9). Upon sensing germinants, typically nutrients, the spore will germinate and outgrow into a vegetative cell (11, 12).

Endospore formation is well characterized in *Bacillus subtilis,* where it affects the expression of over 500 genes (13, 14). Sporulation-associated genes continue to be identified and characterized (15–20). A transposon screen by Meeske et al. (21) identified 157 genes required for the sporulation of *B. subtilis,* including 24 previously unidentified genes. Genes required for sporulation include kinases and phosphotransferases that initiate sporulation and sigma factors that control endospore progression, chromosome segregation, and production of the spore coat. Many of these proteins have dedicated roles in sporulation, while others, such as Spo0A, also play a role in vegetative growth (22–24).

*B. subtilis*, along with many other Bacilli and Clostridia, initiates sporulation via a phosphorelay system (25–27). Five autophosphorylating sensor histidine kinases (KinA-KinE) respond to high cell density and low nutrient availability by transferring a phosphoryl group to Spo0A via the phosphotransferases Spo0F and Spo0B (4, 27–34). Spo0A, the key transcriptional regulator of sporulation, is activated in its phosphorylated form and directly influences the expression of over 100 genes to govern entry into sporulation (35, 36). In Bacilli and Clostridia, sporulation kinases are highly conserved, as is Spo0A, which is found even in non-sporulating members of Bacillota (1). In contrast, many species within Clostridia lack the phosphotransferases, and instead, multiple kinases phosphorylate Spo0A directly (25, 37–39).

*Paenibacillus* is a spore-forming genus of bacteria encompassing over 200 species with diverse characteristics (40). *Paenibacillus* is commonly found in soil, and many species, such as *P. polymyxa*, *P. macerans*, and *P. elgii*, promote the growth of plants through phosphate solubilization or nitrogen fixation (41–43). Species including *P. alvei*, *P. ehimensis*, and *P. kribbensis* contribute to biological control by producing biocidal compounds (44–46). While previously considered non-pathogenic, recent case reports document *Paenibacillus* spp*.,* including *P. alvei* and *P. thiaminolyticus,* to cause sepsis in infants (47–50) and severe infections in adults, typically as an opportunistic pathogen (48, 51–53). *Paenibacillus* is also the etiological agent of American foulbrood, a devastating honeybee brood disease caused by *P. larvae,* for which there is currently no treatment and necessitates burning of infected hives (54, 55). Species such as *P. odorifer*, *P. amylolyticus*, and *P. lactis* are the causative spoilage agents of a variety of food products, including pasteurized and chilled items such as dairy and ready-to-eat meals (56–58). Studying the sporulation of diverse *Paenibacillus* impacts many industries.

Here, we survey 1,460 high-quality *Paenibacillus* genomes for known sporulation-associated protein-coding genes from *B. subtilis*. We detected 632 *B. subtilis* sporulation genes in *Paenibacillus*, with 350 of these genes conserved in ≥95% of the genomes. While much of the sporulation pathway is conserved between *Paenibacillus* and *B. subtilis*, we determined that most *Paenibacillus* encode a unique Spo0B variant containing an N-terminal domain that localizes it to the cell membrane (Spo0B-TM). Spo0B-TM from *P. polymyxa* is not functionally interchangeable with Spo0B in *B. subtilis*, but Spo0B-TM associates with its phosphorelay partners, Spo0F and Spo0A, and the transmembrane domain is likely important for this association. Across Spo0B orthologs in Bacillota, we found that while the phosphorylatable histidine region is strongly conserved in sequence, the rest of the protein is highly variable between species. In the case of Spo0B from *Paenibacillus*, this sequence variation is likely consequential.

## RESULTS

### Survey of the *Paenibacillus* sporulation pangenome identifies core sporulation genes that are shared with other spore-forming Bacillota

To survey the conservation of known sporulation genes throughout the *Paenibacillus* genus, we downloaded all high-quality annotated *Paenibacillus* genome assemblies (*n* = 1,460) from NCBI and performed a BLASTP search using 741 *B. subtilis* sporulation protein sequences. Genes encoding the protein sequences used in our search included genes predicted to be essential for sporulation in *B. subtilis* (21), genes classified by SubtiWiki as sporulation genes (59), and genes in the sporulation gene set defined by Galperin et al. (23). The surveyed *Paenibacillus* genomes represent over 250 unique species (Table S1), with strong representation from *P. polymyxa* (*n* = 117), *P. larvae* (*n* = 64), and *P. odorifer* (*n* = 37). As additional genomes were included in the search, fewer additional sporulation genes were detected, indicating that the search was performed to saturation. Thus, the number and quality of genomes surveyed were sufficient to capture an accurate, extensive representation of likely sporulation genes within the genus that are shared with *B. subtilis*.

**Fig 1:**
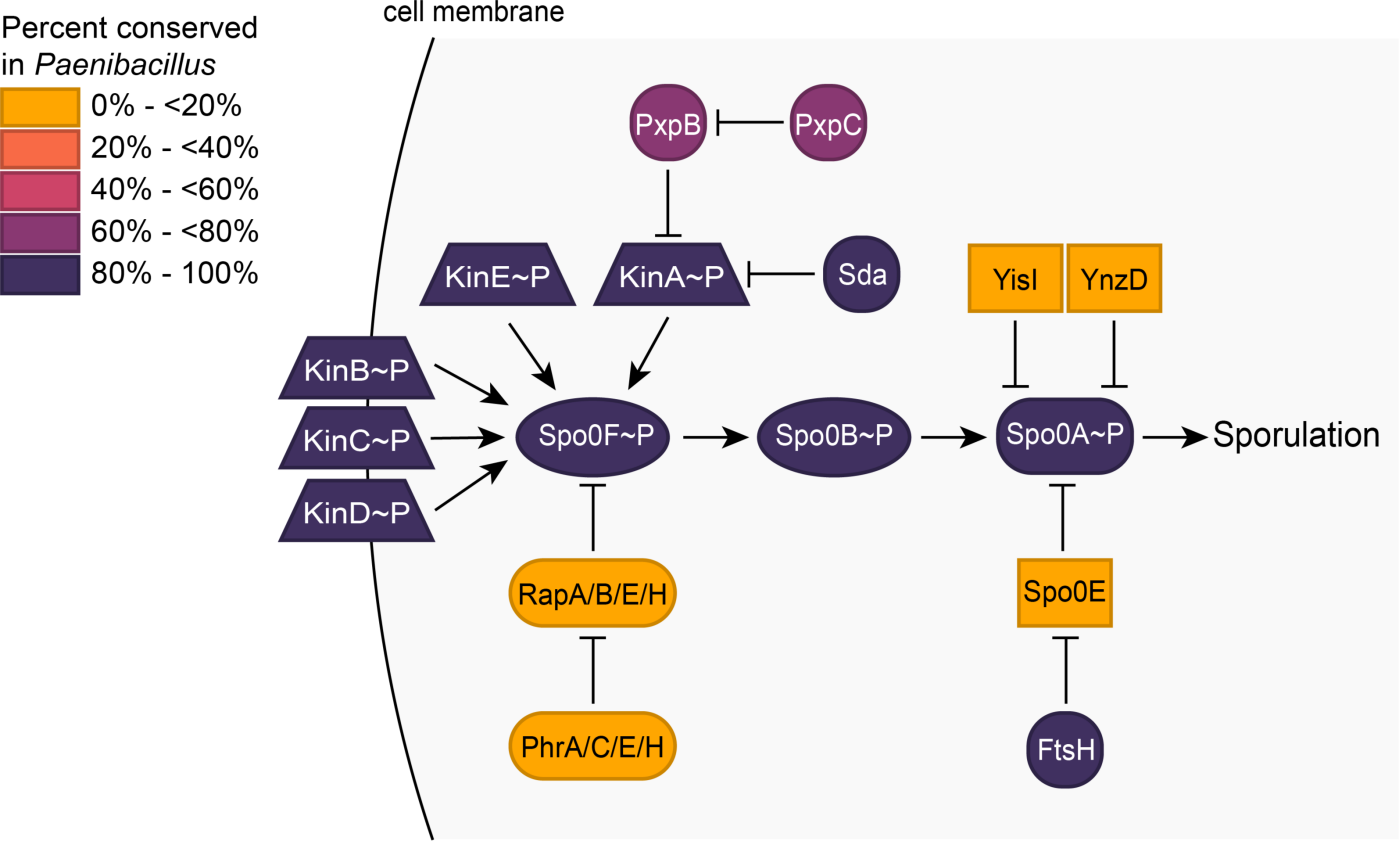
Key sporulation initiation proteins in *B. subtilis* are conserved in *Paenibacillus*. Heat map of the sporulation initiation phosphorelay of *B. subtilis*, showing the conservation of the proteins across *Paenibacillus*. Trapezoids denote kinases; ovals, phosphotransferases; rectangles, phosphatases. Genomes surveyed are listed in Table S1 and a full list of sporulation genes and their conservation can be found in Table S2.

**Fig 2:**
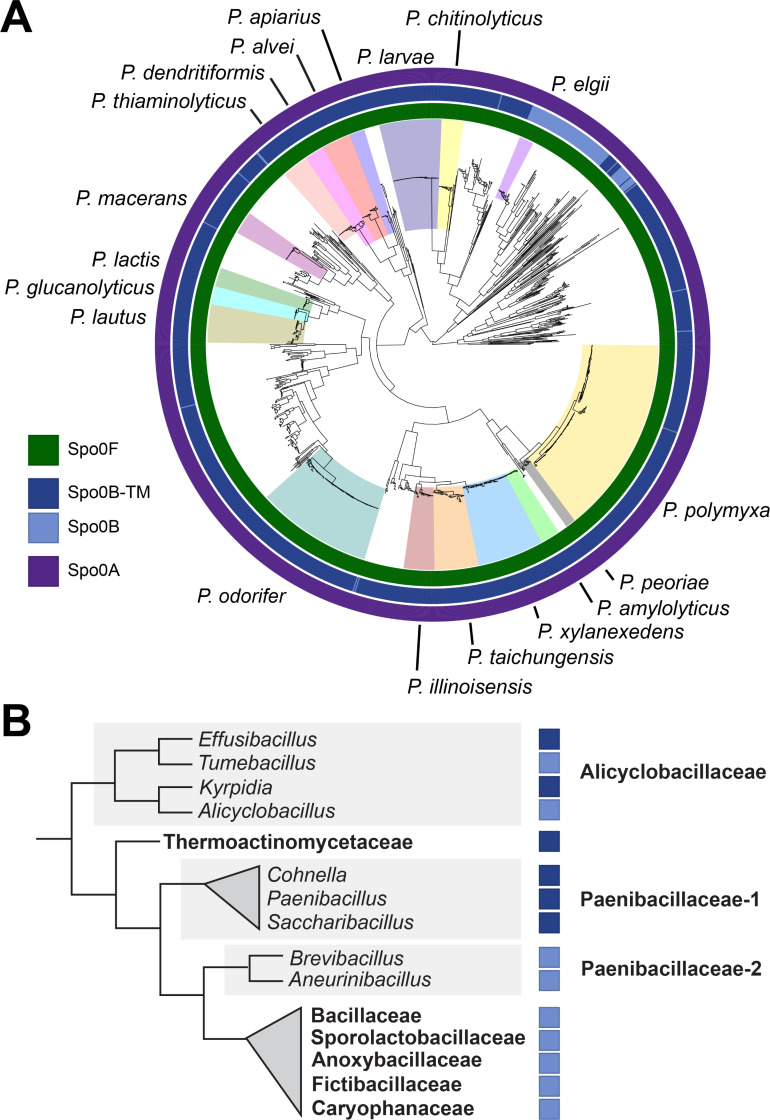
Spo0B has a unique transmembrane domain in most *Paenibacillus* genomes. (**A**) A midpoint-rooted maximum likelihood phylogenetic tree of *Paenibacillus* genomes built with single-copy core genes. Species with at least nine available high-quality genomes are labeled. Color coding indicates the presence of Spo0F, Spo0B, and Spo0A, and the transmembrane domain variant of Spo0B (Spo0B-TM). *Paenibacillus* genomes used in the analysis are listed in Table S1. (**B**) A cladogram of sporulating Bacilli with branching based on the Genome Taxonomy Database (GTDB) (62, 63). Taxonomies are derived from NCBI Taxonomy (64, 63). Families with >10 reference genomes and genera with >5 reference genomes are shown. Family names are bolded, and genera are italicized. When >33% of a family or genus encodes Spo0B-TM, this is indicated with a dark blue box in accordance with the legend in 2A.

Of the 741 *B. subtilis* sporulation genes included in our search, 632 were detected in at least one of the *Paenibacillus* genomes (Table S2), with a mean of 453 sporulation genes detected per genome. To determine which known sporulation genes may be most important for sporulation in *Paenibacillus*, we split the sporulation pangenome into core, shell, and cloud genomes. Genes found in ≥95% of the surveyed genomes made up the core, genes found in <95% but ≥15% of genomes made up the shell, and the remaining genes made up the cloud. Galperin et al. (23) constructed a list of ~120 universally conserved sporulation genes in Bacillota. We detected all 120 of these genes in *Paenibacillus* and found 115 of these genes in the core sporulation genome. The five genes missing from the core genome are *sweC* (detected in 1.4% of genomes), *yciB* (7.8%), *divIC* (28%), *ylbB* (81%), and *yloC* (89%). Over half of the sporulation genes we identified were core genes (*n* = 350), of which 14 are essential for life in *B. subtilis* (60). Meeske et al. (21) identified 142 genes required for sporulation in *B. subtilis,* excluding genes involved in the TCA cycle. We found 127 of these genes in the sporulation core for *Paenibacillus*, and the remaining 15 genes are listed in Table S3. The key players in the phosphorelay, KinA-E, Spo0F, Spo0B, and Spo0A, are conserved in >99% of surveyed *Paenibacillus* genomes (Fig. 1 and 2A; Table S2). The sporulation-specific sigma factors, SigE, SigF, SigK, and SigG (61), are conserved in 100% of the genomes (Table S2). These results indicate that *Paenibacillus* shares a very similar sporulation pathway with *B. subtilis*.

### A Spo0B variant with a predicted transmembrane domain is conserved across *Paenibacillus*

A previous study of *P. polymyxa* sporulation histidine kinases examined the Spo0B sequences of six *Paenibacillus* genomes and found a unique extended N-terminus containing two hydrophobic helices that are predicted to form a transmembrane domain (65). We will refer to Spo0B containing this domain as Spo0B-TM. Of the *Paenibacillus* genomes surveyed, 92% encode Spo0B-TM (Fig. 2A). The remaining 8% of *Paenibacillus* genomes encode a Spo0B that lacks the transmembrane domain. Species that do not contain the transmembrane domain in Spo0B clade together and include *P. elgii*, *P. validus, P. mucilaginosus, P. ehimensis*, and *P. tyrfis* (Fig. 2A).

Due to the strong conservation of Spo0B-TM within *Paenibacillus*, we expanded our search to include all RefSeq reference genomes in the Bacilli class (*n* = 2,425, including 316 *Paenibacillus* spp.). Spo0B was detected in 1,385 genomes, and a predicted transmembrane domain was identified in 27% of Spo0B proteins. This variant is restricted primarily to the clade of Bacilli that includes the families of Paenibacillaceae (78% of 416 species with Spo0B contain the transmembrane domain), Thermoactinomycetaceae (95% of 42 genomes), and Alicyclobacillaceae (38% of 21 genomes) (Fig. 2B). The transmembrane domain is completely absent in clade Paenibacillaceae-2, which contains the genera *Aneurinibacillus* and *Brevibacillus,* and in the clades of Alicyclobacillaceae containing the genera *Tumebacillus* and *Alicyclobacillus* (Fig. 2B). RefSeq accessions and assembly names, taxonomic lineage, and Spo0B and transmembrane domain presence for all Bacilli surveyed are listed in Table S4.

**Fig 3:**
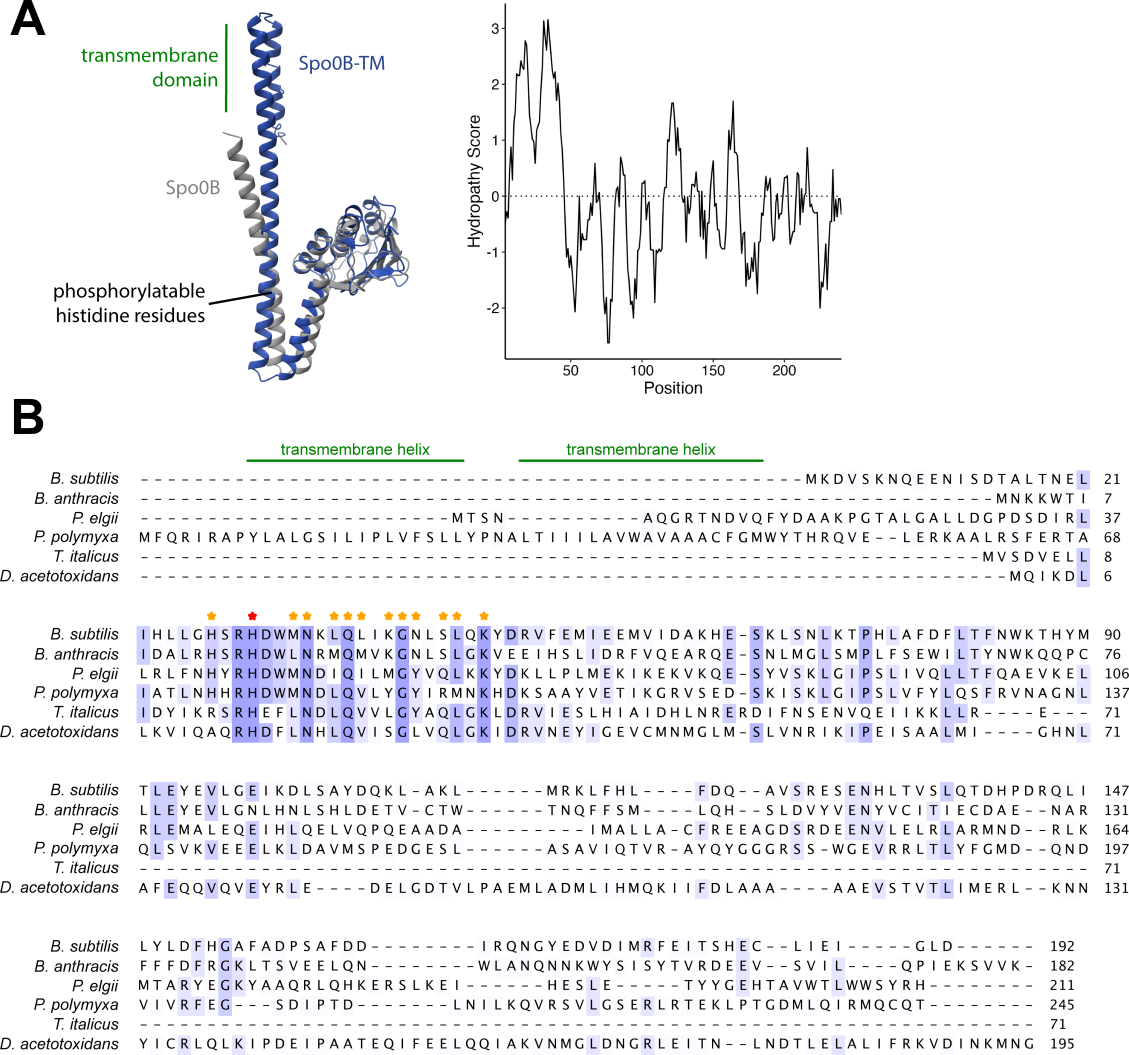
Predicted structure and sequence alignment of Spo0B-TM with other Spo0B proteins from Bacillota. (**A**) AlphaFold model of Spo0B from *B. subtilis* and Spo0B-TM from *P. polymyxa* and Kyte-Doolittle hydropathy plot of Spo0B-TM from *P. polymyxa*. (**B**) Protein sequence alignment of Spo0B in *B. subtilis* and its orthologs across Bacilli (*Bacillus anthracis*, *Paenibacillus elgii*, *Paenibacillus polymyxa*) and Clostridia (*Thermoanaerobacter italicus, Desulfofarcimen acetotoxidans*). Amino acids are shaded based on conservation, with completely conserved residues shown in dark purple. The transmembrane helices of Spo0B-TM from *P. polymyxa* are annotated. Residues in the α1 helix of Spo0B from *B. subtilis* that are known to interact with Spo0F and Spo0A are indicated with stars. The phosphorylatable histidine residue is denoted by the red star. Spo0B sequence accessions are listed in Table S5.

Despite low percent identity at the protein sequence level (19%), Spo0B from *B. subtilis* and Spo0B-TM from *P. polymyxa* are predicted to have a high degree of structural similarity when modeled using AlphaFold (Fig. 3A). Alignment of six Spo0B orthologs from Bacilli and Clostridia revealed a highly conserved region around the phosphorylatable histidine residue (Fig. 3B). As expected, the residues that directly interact with Spo0F and Spo0A in the α1 helix of *B. subtilis* Spo0B (66) are completely or highly conserved (Fig. 3B).

### Spo0B-TM localizes to the cell membrane when expressed in *B*. *subtilis*

**Fig 4:**
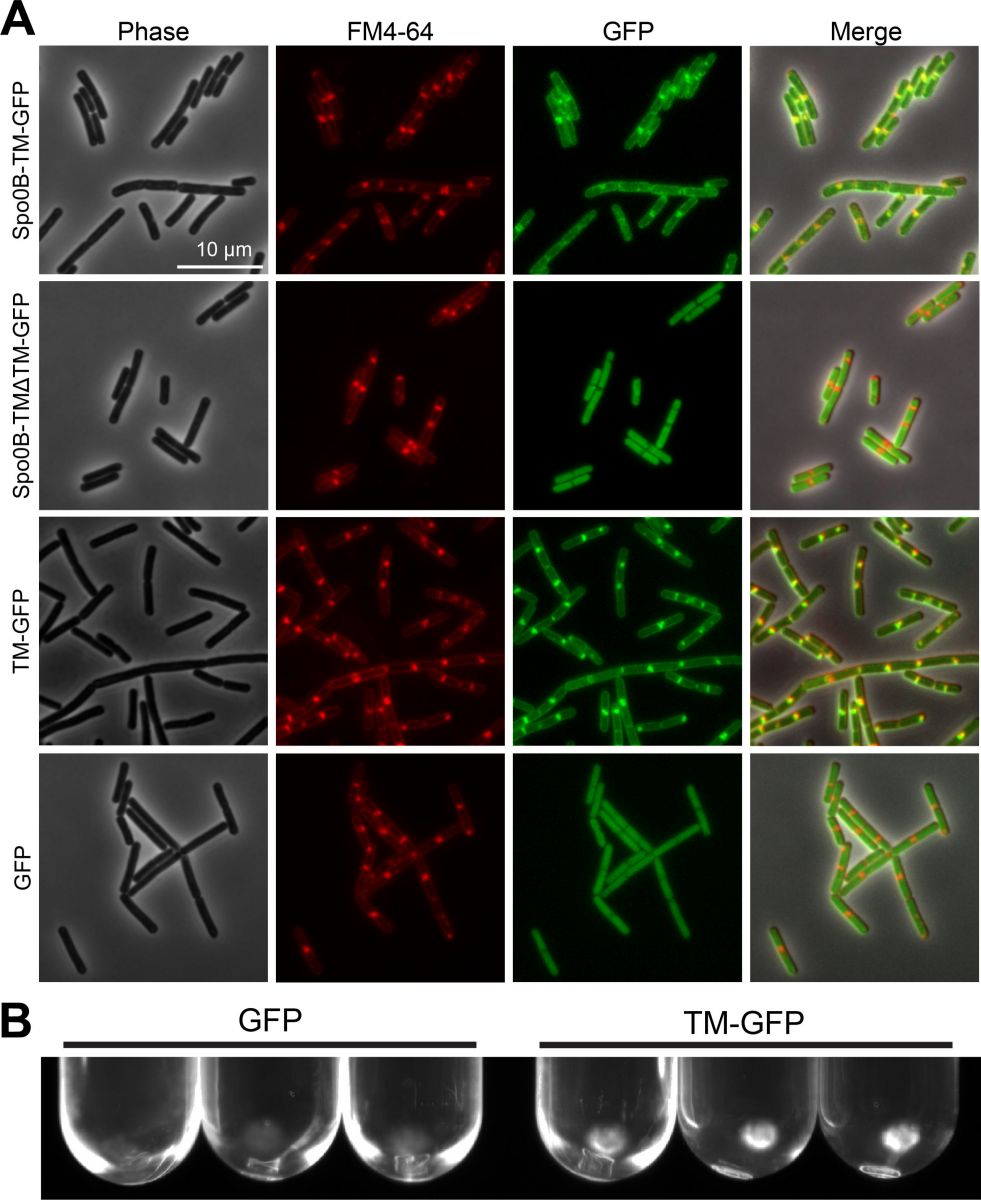
Spo0B-TM localizes to the cell membrane in *B. subtilis*. (**A**) Microscope images of *B. subtilis* expressing *P. polymyxa* Spo0B-TM fused to GFP (Spo0B-TM-GFP) or *P. polymyxa* Spo0B-TM lacking the TM domain fused to GFP (Spo0B-TM∆TM-GFP). TM-GFP indicates the TM domain of Spo0B-TM is fused directly to GFP. Membranes were stained with FM4-64. (**B**) Pellets containing membrane fractions illuminated by blue light.

To determine whether the bioinformatically predicted transmembrane domain of Spo0B-TM imparts membrane localization, we fused GFP to the C-terminus of *P. polymyxa* Spo0B-TM (Spo0B-TM-GFP) and expressed this protein in *B. subtilis*. As a control, we fused GFP to the C-terminus of *P. polymyxa* Spo0B-TM from which we deleted the transmembrane domain (Spo0B-TM∆TM-GFP). We then monitored the cellular localization of these proteins using fluorescence microscopy. Spo0B-TM-GFP localized to the cell membrane, exhibiting a membrane localization pattern that was similar to the membrane stain FM4-64 (Fig. 4A). In contrast, Spo0B-TM∆TM-GFP was located throughout the cytoplasm. To further confirm that the transmembrane domain is sufficient to impart localization, we fused only the transmembrane domain to GFP (referred to as TM-GFP). TM-GFP visibly localizes to the membrane, similar to Spo0B-TM-GFP. In contrast, GFP lacking the transmembrane domain is diffuse throughout the cytoplasm (Fig. 4A).

To further confirm membrane localization of the transmembrane domain from Spo0B, we used a biochemical approach to fractionate the cell membrane (67). Consistent with the results of the microscopy, pellets resulting from ultracentrifugation of cell lysate collected from *B. subtilis* cells expressing TM-GFP fluoresced under blue light, whereas pellets from *B. subtilis* cells expressing GFP without the transmembrane domain did not fluoresce (Fig. 4B). Collectively, our results indicate that Spo0B-TM from *P. polymyxa* exhibits membrane localization and that the transmembrane domain alone is sufficient for localization.

### Spo0B-TM interacts with phosphorelay proteins Spo0A and Spo0F from *P. polymyxa*

**Fig 5:**
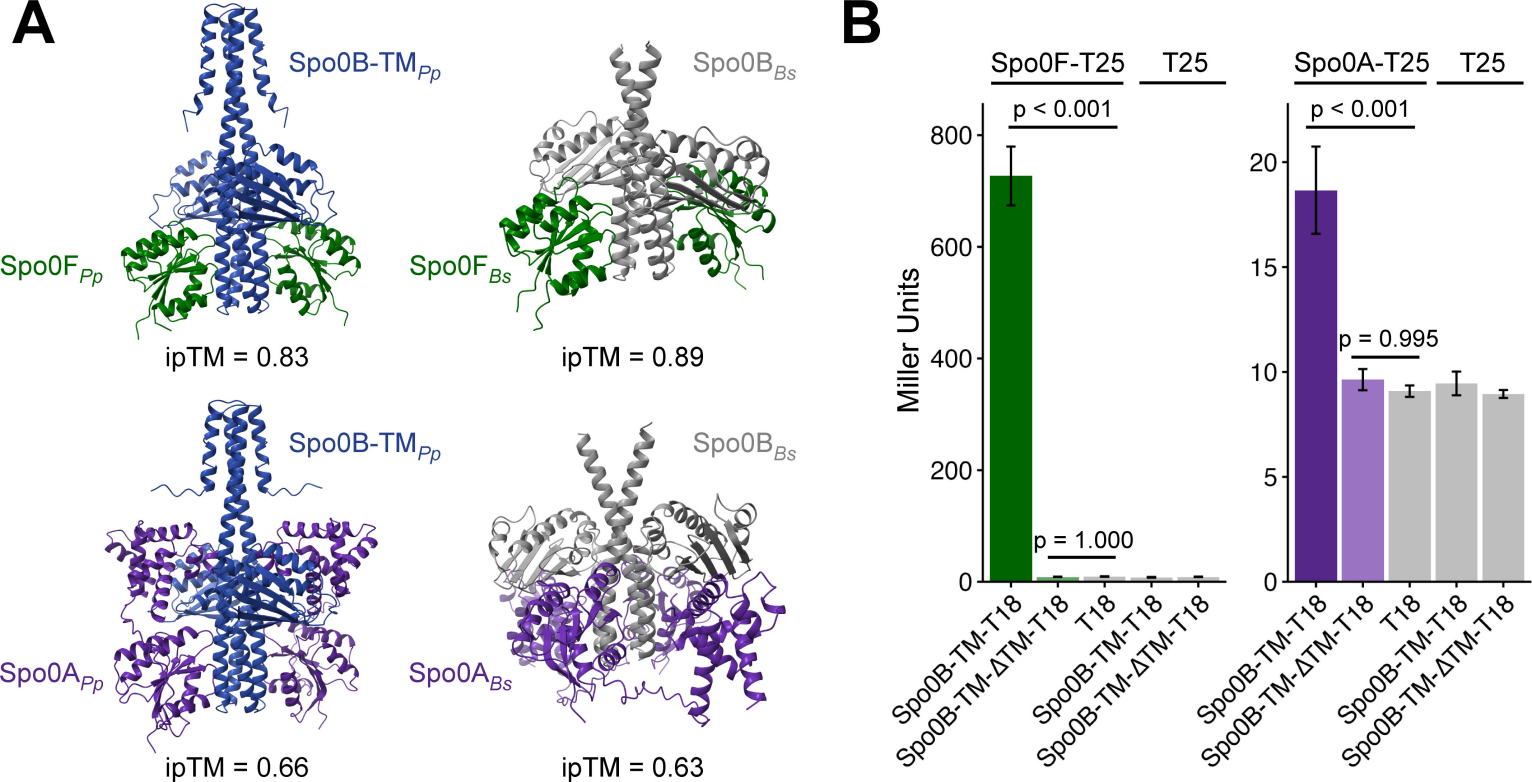
Spo0B-TM interacts with both Spo0F and Spo0A from *P. polymyxa*. (**A**) AlphaFold models of *P. polymyxa* and *B. subtilis* protein interactions. ipTM scores >0.8 represent high confidence predictions. *Pp, P. polymyxa; Bs, B. subtilis*. Protein sequence accessions used for modeling are listed in Table S5. (**B**) Quantification of β-galactosidase activity for BACTH constructs using *P. polymyxa* proteins, representative of nine biological replicates. Error bars represent SEM. *P*-values indicate the results of a one-way ANOVA followed by Tukey’s test.

To predict if *P. polymyxa* Spo0F and Spo0A could interact with Spo0B-TM, we modeled the interactions using AlphaFold 3 (Fig. 5A). The interaction between Spo0F and Spo0B-TM had an ipTM score >0.8, indicating a confident, high-quality prediction (68). The interaction between Spo0A and Spo0B-TM had an ipTM score of 0.66, indicating the prediction was made with lower confidence. However, the interaction model of *B. subtilis* Spo0A and Spo0B, proteins known to interact, similarly yielded an ipTM score of 0.63. Therefore, the low score of the Spo0A and Spo0B-TM interaction should not discredit the modeled interaction. The location of the binding between the two protein pairs across species also appeared similar (Fig. 5A).

We then examined the *in vivo* interaction of Spo0B-TM with Spo0A and Spo0F from *P. polymyxa* using the bacterial adenylate cyclase two-hybrid (BACTH) system in *Escherichia coli*. The BACTH system utilizes the interaction-mediated reconstitution of adenylate cyclase from *Bordetella pertussis* (69). The catalytic domain of this protein is active only when its two complementary fragments (T18 and T25) are brought together to allow for functional complementation. When interacting proteins are fused to T18 and T25, heterodimerization of the proteins results in functional complementation of the adenylate cyclase fragments. Adenylate cyclase synthesizes cAMP, which induces the production of β-galactosidase in the cell. Therefore, we used β-galactosidase activity as a reporter to quantify protein-protein interactions.

We fused *P. polymyxa* Spo0F or Spo0A to the T25 fragment and *P. polymyxa* Spo0B-TM to the T18 fragment of the adenylate cyclase catalytic domain. Spo0B-TM showed strong interaction with Spo0F, with a β-galactosidase activity of 727 ± 53 Miller units (Fig. 5B). The interaction of Spo0B-TM with Spo0A yielded a more modest level of β-galactosidase activity (19 ± 2 Miller units), but this activity was significantly higher than the negative controls (*P* < 0.001 compared with all negative controls) (Fig. 5B). Interestingly, Spo0B-TM did not exhibit detectable interaction with either Spo0A or Spo0F when the transmembrane domain was deleted (Spo0B-TM∆TM), with β-galactosidase activity comparable to the negative controls (Fig. 5B). These results suggest that Spo0B-TM interacts with Spo0A and Spo0F of *P. polymyxa*, and that the transmembrane domain is necessary to detect this interaction. One caveat of the BACTH system is that protein levels of Spo0B-TMΔTM-T18 may be reduced compared with Spo0B-TM-T18. In either case, these data suggest that the TM domain of Spo0B has an important role in the function of this protein.

### Spo0B-TM is syntenic, but not functionally interchangeable with Spo0B in *B. subtilis*

**Fig 6:**
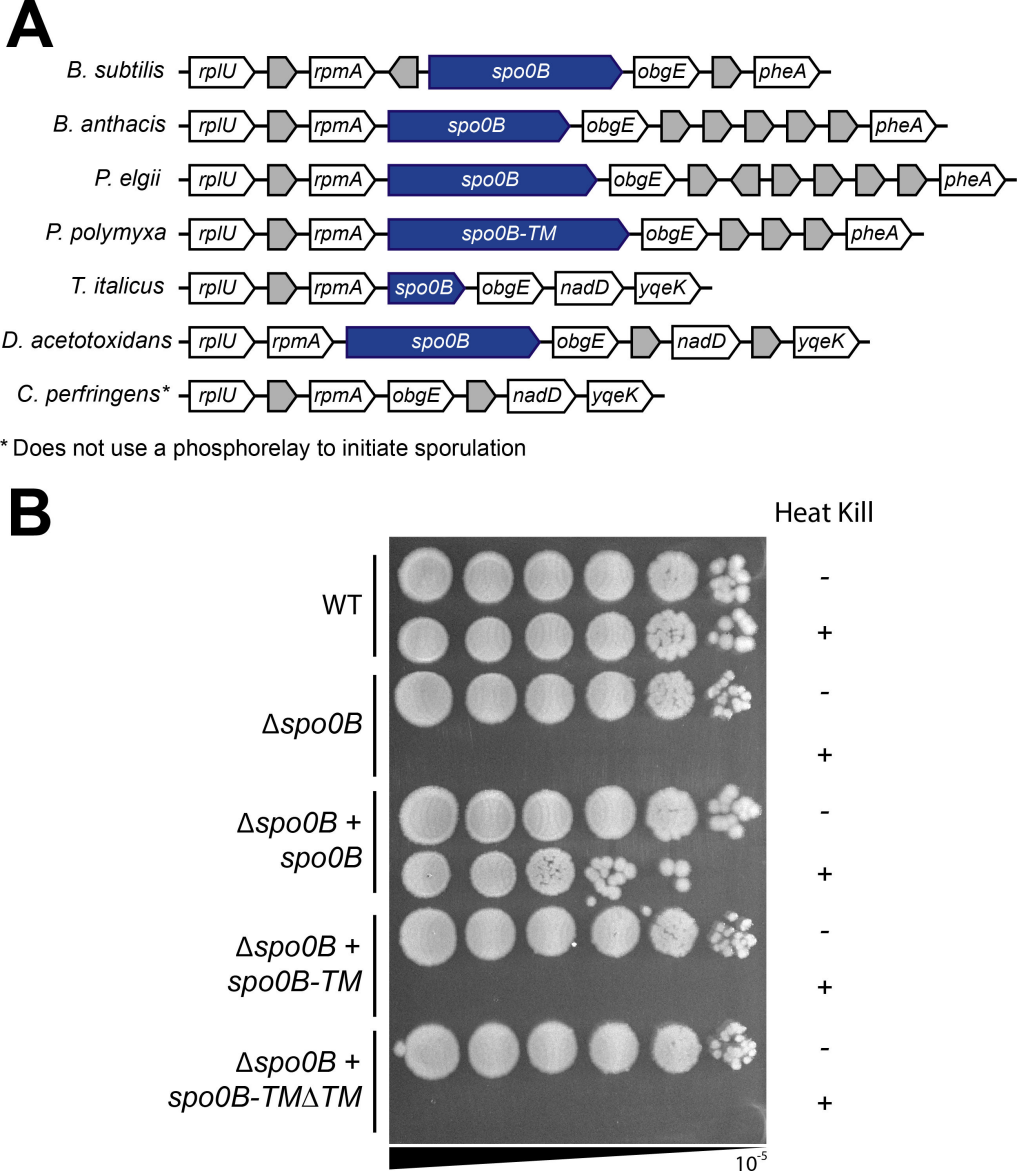
Spo0B-TM from *P. polymyxa* does not rescue the sporulation defect of a *spo0B* deletion in *B. subtilis*. (**A**) Synteny diagram of the *spo0B* genomic region across *Bacillus subtilis, Bacillus anthracis, Paenibacillus elgii* (*spo0B*), *Paenibacillus polymyxa* (*spo0B-TM*), *Thermoanaerobacter italicus, Desulfofarcimen acetotoxidans,* and *Clostridium perfringens*. Genome accessions are listed in Table S5. (**B**) Sporulation assay of *B. subtilis* strains. Serial dilutions of culture were plated before and after heating to 80˚C to kill vegetative cells. Spot titers are representative of three biological replicates.

While two-component signaling proteins are typically organized in pairs or in adjacent operons (70), the genes encoding the sporulation initiation phosphorelay are scattered throughout the genome. In *B. subtilis*, *spo0B* is located downstream of *rplU* and *rpmE*, genes encoding ribosomal proteins bL21 and bL31 (71, 72), and upstream of *obg*, the gene encoding the GTP-binding protein Obg (73). We examined the syntenic regions in Bacilli and Clostridia species and found that the genomic arrangement of *spo0B* to be well conserved (Fig. 6A).

Since the genomic context and the phosphorylatable histidine domain of Spo0B-TM are conserved and Spo0B-TM can interact with other members of the *P. polymyxa* phosphorelay, we hypothesized that Spo0B-TM may be functional in related spore-formers. To test if *spo0B-TM* is functionally interchangeable with *spo0B* in *B. subtilis*, we expressed *P. polymyxa spo0B-TM* under the control of the native *spo0B* promoter and determined whether it could complement the *Δspo0B* mutant of *B. subtilis*. Spo0B is essential for sporulation in *B. subtilis*, and as expected, the *∆spo0B* mutant failed to produce spores (Fig. 6B). Complementation with *spo0B* expressed under its native promoter restored sporulation efficiency to nearly wild-type levels (Fig. 6B). The incomplete complementation may be because deleting *spo0B* has a polar effect on the downstream gene *obg*, a gene that is essential for both growth and sporulation (74, 75). Complementation with *spo0B-TM*, regardless of the presence or absence of its transmembrane domain, did not rescue the sporulation defect of the *∆spo0B* mutant, as no heat-resistant spores were detected in either strain (Fig. 6B). Therefore, *spo0B-TM* and *spo0B-TM∆TM* from *P. polymyxa* are not functionally interchangeable with *spo0B* in *B. subtilis*, despite the highly conserved region surrounding the phosphorylatable histidine.

### Spo0B exhibits the lowest sequence identity of members of the phosphorelay

**Fig 7:**
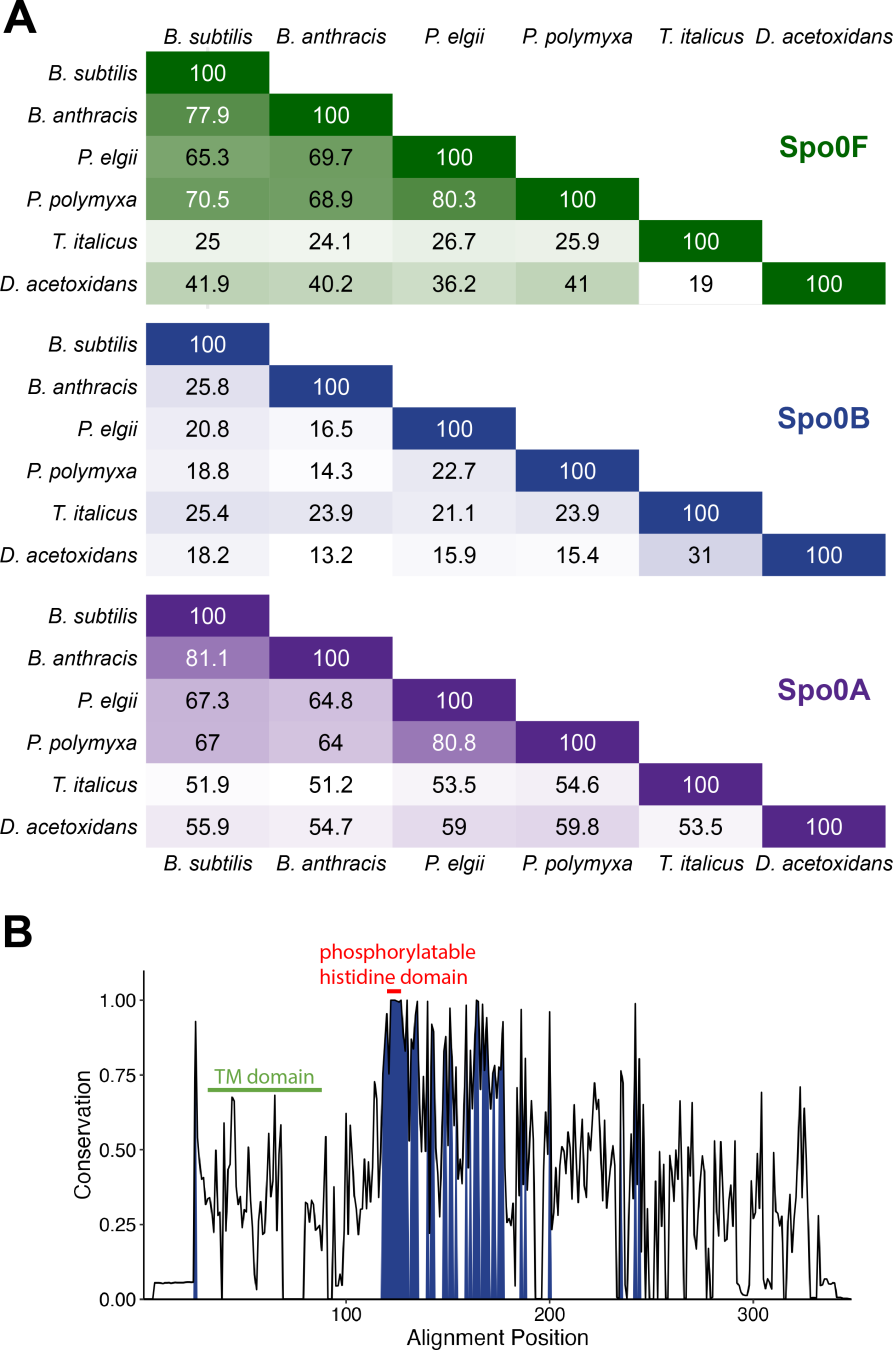
Spo0B protein sequences are not well conserved. (**A**) Pairwise percent identity matrix of Spo0F, Spo0B, and Spo0A orthologs, calculated using Clustal Omega. (**B**) Protein sequence conservation plot of Spo0B-TM and Spo0B from *Paenibacillus* (*n* = 1,437). Residues shared by ≥75% of sequences are shaded dark blue. Features are labeled with respect to the Spo0B-TM sequence from the reference genome of *P. polymyxa*. Protein sequence accessions are listed in Table S5.

Since neither Spo0B-TM nor Spo0B-TM∆TM from *Paenibacillus* could rescue sporulation in a *∆spo0B* mutant of *B. subtilis*, we next examined the sequence conservation of Spo0B proteins across Bacillota. Despite a high degree of structural similarity predicted by AlphaFold (Fig. 3A), *B. subtilis* and *P. polymyxa* Spo0B share only 19% protein sequence identity (Fig. 7A). In contrast, Spo0F and Spo0A from these species share 70% and 67% protein sequence identity, respectively. Consistent with the low sequence identity of Spo0B, a search using BLASTN with *B. subtilis spo0B* as the query identified no significant Spo0B homologs in *Paenibacillus*. However, a search with BLASTP yielded hits for Spo0B in 1,450/1,460 genomes. Using HMMER, we were able to identify Spo0B in all but one *Paenibacillus* genome when we used the protein sequences of Spo0B from *P. polymyxa* and *P. validus* as the query.

The sequence of Spo0B is known to be poorly conserved among *Bacillus* species (76, 77), and we determined this to be true among *Paenibacillus* species as well (Fig. 7B). In an alignment of 1,437 Spo0B-TM and Spo0B sequences from *Paenibacillus* (length = 246 ± 12 amino acids), six residues were 100% conserved (Fig. 7B). Five of these residues are part of the highly conserved region around the phosphorylatable histidine residue. Only 46 residues were conserved in >75% of sequences (Fig. 7B). The sequence of the transmembrane domain was not strongly conserved, although its hydrophobic properties are maintained, suggesting that the importance of this domain is membrane localization. Altogether, these data suggest that the phosphorylatable histidine domain is the most fixed feature of this protein, while the remaining sequence can evolve more freely.

## DISCUSSION

Bacillota diversified from other phyla very early in evolutionary history (78), and endospore formation is likely an ancestral trait of this phylum. Thus, the ability to form spores encompasses diverse bacteria that inhabit a broad range of environments. Sporulation genes have largely been inherited vertically, with approximately 120 genes shared across endospore formers within Bacillota (23). Although previous studies included single representatives of *Paenibacillus*, we detected most of these genes in our expanded survey, which provides further support for their universal conservation. Core genes are involved in all stages of sporulation from initiation to germination, indicating that the fundamental process of sporulation is shared in most Bacillota. Nevertheless, even within this core sporulation genome, there is unexpected diversity that likely reflects differences in environmental contexts and signals triggering the decision to sporulate. The *Paenibacillus* genus was originally classified within Bacillaceae but has been subsequently reclassified (63). Our analysis of sporulation gene conservation across *Paenibacillus* spp. may aid in further resolution of their phylogenetic relationships.

The ancestral sporulation initiation pathway is hypothesized to have been a phosphorelay (25), in which the phosphotransferases Spo0F and Spo0B aid in the transfer of the phosphoryl group from a histidine kinase to Spo0A (Fig. 1). Spo0B, a cytoplasmic dimeric histidine transferase, may have evolved from a histidine kinase that lost its N-terminal signal detection domain and C-terminal ATP-binding domain (79). Spo0B ortholog sequences tend to vary greatly, even between species in the same genus (65, 76, 77) (Fig. 3B and 7). One of the unusual features of Spo0B identified in *Paenibacillus* is an N-terminal extension predicted by Park et al. (65) to encode a transmembrane domain. This domain is absent from Spo0B in *B. subtilis*. Park and colleagues found this domain in Spo0B sequences in a total of six *Paenibacillus* genomes. We confirmed that this predicted transmembrane domain does indeed facilitate membrane localization of *P. polymyxa* Spo0B-TM (Fig. 4). We identified Spo0B in all but one of the *Paenibacillus* genomes we surveyed (*n* = 1,460), and of these, 92% encode Spo0B-TM (Fig. 2A). Outside of *Paenibacillus* spp., we detected Spo0B-TM in other species in the Paenibacillaceae family and even in the neighboring families Thermoactinomycetaceae and Alicyclobacillaceae (Fig. 2B). The high degree of conservation in these families suggests that Spo0B-TM was present in their common ancestor, and the transmembrane domain was subsequently lost in certain lineages.

What is the importance of the transmembrane domain of Spo0B in *Paenibacillus*? Species from *Paenibacillus* lineages that encode Spo0B without the transmembrane domain have been experimentally shown to sporulate (80–82), which suggests that Spo0B lacking the transmembrane domain is functional in these lineages. Nevertheless, the high conservation of the transmembrane domain suggests it imparts a strong selective advantage in this genus. One possibility is that *Paenibacillus* may rely more heavily on the membrane-localized kinases (KinB, KinC, and KinD) than the cytoplasmic kinases (KinA and KinE) to initiate sporulation. *B. subtilis* is more dependent on KinB for sporulation in minimal media (83). KinA-E are highly conserved in *Paenibacillus* (Fig. 1; Table S2). If there are environmental conditions experienced by *Paenibacillus* that increase reliance on membrane-localized kinases to initiate sporulation, then it may be advantageous to localize a member of the phosphorelay to the membrane as well.

The transmembrane domain of Spo0B-TM may also enhance its interaction with Spo0A and Spo0F. Using a BACTH assay, we found that deleting the transmembrane domain eliminated detectable interaction with both Spo0A and Spo0F of *P. polymyxa* (Fig. 5B). In *B. subtilis*, Spo0B forms dimers through the interaction of the N-terminal helical domains, and dimerization is essential to form the four-helix bundle interaction site necessary for phosphoryl transfer (84). Therefore, the additional helices supplied by the transmembrane domain could help to stabilize dimer formation. Increased dimerization may enable detection by the BACTH assay, either through increased stabilization of the Spo0B protein itself or through stabilization of binding interactions with its phosphorelay partners.

Among members of the sporulation initiation phosphorelay, we found that Spo0B exhibits the most sequence diversity (Fig. 7A). The phosphorelay is an expanded version of a typical two-component system, and the interaction surface residues of the proteins in these systems resist evolutionary change. For example, 20 out of 21 interaction residues of Spo0F in *B. subtilis, Bacillus anthracis,* and *Bacillus halodurans* are identical, while only 50% of the remaining residues are identical (85). Accordingly, the most well-conserved region of Spo0B is the region surrounding the phosphorylatable histidine residue (Fig. 3B). Spo0B orthologs have a much lower sequence conservation, and in some instances, have even acquired additional function. Spo0B in *B. anthracis* has pleiotropic functions, including phosphotransferase, autophosphorylation, and ATPase activity as a result of having acquired ATP-binding and hydrolysis domains (77). The physiological consequence of gaining ATPase function has not been determined, but it has been proposed that this expanded functionality could be important for vegetative growth or even pathogenesis.

Our study highlights the importance of studying sporulation proteins in non-model organisms, since variations can be identified even in highly conserved sporulation genes. *Paenibacillus* species inhabit diverse environments and cause a broad range of industrial and public health impacts, from milk spoilage to beehive collapse to human infections. And yet, the majority of what we know about endospore formation comes from *B. subtilis*, mainly due to its genetic tractability. The growing number of *Paenibacillus* genomes available will greatly facilitate future surveys of this kind. Moreover, the wealth of environmental isolates that can be obtained from environmental sources makes *Paenibacillus* an attractive and underappreciated model of sporulation with important industrial and clinical applications.

## MATERIALS AND METHODS

### Strains and media

*B. subtilis* strains were derived from 168 *trpC2* (86) and grown in Lysogeny Broth (LB) at 37°C with aeration. Genomic DNA from the *∆spo0B::kan* strain from the BKK collection (87) was used to create the ∆*spo0B* strains by transformation into 168 *trpC2*. Plasmids used can be found in Table 1, and novel plasmid sequences are available on the project GitHub at https://github.com/isabella-n-lin/paenibacillus_sporulation/blob/main/paeni_plasmid_seqs.fasta. Antibiotics were used at final concentrations of 100 µg/mL ampicillin, 50 µg/mL kanamycin, and 100 µg/mL spectinomycin. *P*. *polymyxa* ATCC 842 was obtained from the American Type Culture Collection and grown in Brain Heart Infusion (BHI) broth at 30°C with aeration. All experiments were performed with three biological replicates, unless otherwise stated.

**TABLE 1:** Strains, plasmids, and primers

	Description or sequence	Source or reference
**Strain**		
BTH101	*E. coli F*^*-*^, *cya*-99, *araD139*, *galE15*, *galK16*, *rpsL1* (*Str*^*r*^), *hsdR2*, *mcrA1*, *mcrB1*	(69)
HAF1	*B. subtilis* wild type 168 *trpC2*	(86)
IM39	*P. polymyxa* ATCC 842	(88)
CP347	168 *trpC2* pHF549 *amyE::P*_*xylA*_*-GFP-3xFLAG*	This study
CP348	168 *trpC2* pHF549 *amyE::P*_*xylA*_*-TM-GFP-3xFLAG*	This study
IM37	168 *trpC2 ∆spo0B::kan*	This study
IM71	168 *trpC2 P*_*spo0B*_*-spo0B::amyE ∆spo0B::kan*	This study
IM72	168 *trpC2 P*_*spo0B*_*-spo0B-TM::amyE ∆spo0B::kan*	This study
IM73	168 *trpC2 P_spo0B_-spo0B-TM∆TM::amyE ∆spo0B::kan*	This study
**Plasmid**		
pHF549	pDR111 with P*_xylA_*, amp^R^, spec^R^. Integration at *amyE*	This study
pCP336	pHF549 *P_xylA_-GFP-3xFLAG*	This study
pCP338	pHF549 *P_xylA_-TM-GFP-3xFLAG*	This study
pKNT25	BACTH plasmid *cyaA* T25 fragment N-terminal fusion	(69)
pUT18	BACTH plasmid *cyaA* T18 fragment N-terminal fusion	(69)
pIM95	pKNT25 carrying *spo0F* from *P. polymyxa*	This study
pIM79	pUT18 carrying *spo0B-TM* from *P. polymyxa*	This study
pIM75	pUT18 carrying *spo0B-TM∆TM* from *P. polymyxa*	This study
pIM92	pKNT25 carrying *spo0A* from *P. polymyxa*	This study
**Primer**		
IM11-F	5′-CTGCAGGTCGACTCTAGAGTTCCAGCGGATAAGAGCGCC-3′	
IM12-R	5′-TGAATTCGAGCTCGGTACCATTGTCTGGCACTGCATTCGAATTTG-3′	
IM13-F	5′-CTGCAGGTCGACTCTAGAGTGGTATACCCATAGACAGGTTGAACTTGAG-3′	
IM14-F	5′-TGCCTGCAGGTCGACTCTAGAGCAAAAAATTGAGGTATTGTTGGCTGACGAC-3′	
IM15-R	5′-TTGAATTCGAGCTCGGTACCGTGGACACCTTATTCTCAATCCGCAATT-3′	
IM18-F	5′-GCCTGCAGGTCGACTCTAGAGGAAAAGAAAAAAGTATTAATCGTCGATGACCAGAAT-3′	
IM19-R	5′-TTGAATTCGAGCTCGGTACCAAGTCACCTCTGAGTTGTTTGTTCACGG-3′	

### *Paenibacillus* sporulation pangenome and phylogenetic tree building

All available *Paenibacillus* genome assemblies as of 21 April 2025 were downloaded from the NCBI Reference Sequence (RefSeq) collection as annotated by the NCBI Prokaryotic Genome Annotation Pipeline (PGAP) (89). Genomes with CheckM (90) contamination greater than 5% or completeness less than 95% were removed. Any genomes suppressed by NCBI by 28 July 2025 were removed, resulting in 1,460 genomes. Genome assembly numbers can be found in Table S1. The phylogenetic tree was made with GToTree v1.8.14 (91), using the prepackaged single-copy gene set for bacteria (74 target genes). The tree was visualized using the ggtree v3.10.1 package (92) and midpoint rooted using the phangorn v2.12.1 package (93). A comprehensive list of sporulation genes was compiled from previous work (21, 23) and SubtiWiki (59) (Table S2). *B. subtilis* sporulation protein sequences were obtained from SubtiWiki (59). Sporulation genes were detected in *Paenibacillus* genomes using BLASTP v2.16.0 (94) and an E-value cutoff of 0.05.

### Spo0B search and transmembrane-domain prediction

HMMER v3.4 (phmmer) (95) was used to search for Spo0B across all proteins annotated by the NCBI PGAP pipeline in the previously described *Paenibacillus* data set (Table S1) and all reference genomes in the class of Bacilli from the NCBI RefSeq database (downloaded on 5 November 2025) (Table S4). Spo0B sequences from *P. polymyxa* and *P. validus* were used as the phmmer query for *Paenibacillus* spp., and Spo0B sequences from *P. polymyxa*, *P. validus*, *B. subtilis,* and *Brevibacillus brevis* were used as the query for Bacilli. Spo0B protein accessions for the queries can be found in Table S5. DeepTMHMM v1.0.42 (96) was used to predict transmembrane domains.

### Spo0F, Spo0B, and Spo0A sequence analysis and structure modeling

The Kyte-Doolittle hydropathy plot was created using ProtScale on the ExPASy server (97) using the Spo0B sequence WP_019688407.1. AlphaFold models were created using AlphaFold 3 (98) and visualized using ChimeraX v1.9 (99). Protein sequences of Spo0F, Spo0B, and Spo0A were obtained from NCBI (100), and the accession numbers can be found in Table S5. Percent identity was calculated using Clustal Omega v1.2.4 (101). The alignment in Fig. 3B was made using Clustal Omega v1.2.4 (101) and visualized using the pyMSAviz v0.5.0 package (102). The conservation plot in Fig. 7B was created using the bio3d v.2.4-5 package (103). Spo0B sequences for the conservation plot were aligned using MAFFT v7.520 (104), using a gap opening penalty of 3 and gap extension penalty of 0.3.

### Membrane localization microscopy

*B. subtilis* overnight cultures were normalized to an OD600 of 0.05 and grown to an OD of 1.5 at 37°C with aeration in LB with 1% xylose for induction of GFP-tagged proteins. For microscopy, cells were harvested, washed with 1× phosphate-buffered saline, and stained with fluorescent dye FM4-64 (Invitrogen T3166) for 5 min at room temperature. Stained cells were mounted on 1% agarose pads for imaging. Microscopy was performed with a Nikon ECLIPSE Ni-E upright microscope with GFP-FITC and RFP-TRITC filter cubes and a Prime SBI Express camera. Exposure times for GFP, FM4-64, and phase microscopy were 700, 100, and 26 ms, respectively. Images were processed using ImageJ v1.53k (105). GFP fluorescence is autoscaled in each image.

### Membrane fractionation

*B. subtilis* overnight cultures were normalized to an OD600 of 0.05 and grown to an OD of 1.5 at 37°C with aeration in LB with 1% xylose for induction of GFP-tagged proteins. Cells were centrifuged at 10,000 × *g* for 15 min at room temperature (Sorvall LYNX 4000, Fiberlite F12-6x500 LEX rotor), resuspended in Buffer A (50 mM Tris-HCl, pH 7.5, 150 mM NaCl, 5 mM MgCl2, 10% glycerol v/v), and flash-frozen by dripping into liquid nitrogen. Cells were lysed by cryomilling at 15 Hz for 3 min, repeated 3 times (Retsch Mixer Mill MM 400), and lysates were clarified by centrifuging at 10,000 rpm for 10 min at 4°C (Beckman Coulter Avanti J-15R, rotor JA-10.100). To pellet membranes and their associated proteins, clarified lysates were centrifuged at 45,000 rpm for 1 h at 4°C (Beckman Coulter Optima XE-90 Ultracentrifuge, TI70 rotor). Membrane pellets were visualized using a BioRad ChemiDoc Imaging System and the Alexa Fluor 488 setting.

### Bacterial two-hybrid system

To investigate the protein interactions of Spo0B-TM from *P. polymyxa*, we used the commercially available BACTH System Kit from Euromedex. Strains were derived from the non-reverting adenylate cyclase-deficient *E. coli* strain BTH101. Plasmid gene inserts were amplified from *P. polymyxa* ATCC 842. All proteins of interest were C-terminally tagged with either T18 or T25 fragments.

For the β-galactosidase assay, strains were grown in LB supplemented with ampicillin, kanamycin, and 1% glucose for 6 h at 30°C. The cultures were used to inoculate LB supplemented with ampicillin, kanamycin, and 0.5 mM IPTG and grown for 18 h at 30°C. The cells were lysed using lysis buffer (100 mL Z-buffer (0.06M Na_2_HPO_4_, 0.04M NaH_2_PO_4_-H_2_O, 0.01M KCl, 0.001M MgSO_4_-7H_2_O), 270 μL β-mercaptoethanol, 50 μL 10% sodium dodecyl sulfate) and 20 μL chloroform and incubated at 30°C. The reaction was started upon the addition of o-nitrophenyl-β-D-galactopyranoside (4 mg/mL in Z-buffer) to the lysed cells. The reaction was stopped with 1M Na_2_CO_3_ when a pale yellow color developed. Absorbances at 600, 420, and 550 nm were measured with a BioTek Synergy H1 microplate reader, Gen 5 3.11, for cell density, o-nitrophenol production, and background, respectively. β-galactosidase activity was measured in Miller units. *P*-values were calculated with the R stats v4.3.2 package using a one-way ANOVA followed by Tukey’s test. Experiments were performed using nine biological replicates.

### Sporulation assays

Sporulation was induced by nutrient exhaustion in Difco sporulation medium (DSM). Strains were grown in DSM on a roller drum at 37°C for 3 h, then normalized to OD600 of 0.05 and incubated at 37°C, shaking at 220 rpm for 24 h. The cultures were serially diluted in Tbase + 1 mM MgSO_4_ and plated on LB agar plates before and after heating for 20 min at 80°C.

## Data Availability

Genome and protein accessions can be found in Tables S1, S4, and S5. Scripts for data acquisition and analyses are available on GitHub at https://github.com/isabella-n-lin/paenibacillus_sporulation.

## SUPPLEMENTAL MATERIAL

Tables S1 to S5 jb.00367-25-s0001.xlsx Genome accession numbers for all genomes used in all figures.

Supplemental table legends jb.00367-25-s0002.docx Legends for Tables S1 to S5.

ASM does not own the copyrights to Supplemental Material that may be linked to, or accessed through, an article. The authors have granted ASM a non-exclusive, world-wide license to publish the Supplemental Material files. Please contact the corresponding author directly for reuse.

## References

[1] Galperin MY. 2013. Genome diversity of spore-forming firmicutes. Microbiol Spectr 1:TBS–0015 doi:10.1128/microbiolspectrum.TBS-0015-2012 26184964 PMC4306282

[2] Yutin N, Galperin MY. 2013. A genomic update on clostridial phylogeny: gram-negative spore formers and other misplaced clostridia. Environ Microbiol 15:2631–2641. doi:10.1111/1462-2920.12173 23834245 PMC4056668

[3] Setlow P, Christie G. 2023. New thoughts on an old topic: secrets of bacterial spore resistance slowly being revealed. Microbiol Mol Biol Rev 87:e0008022. doi:10.1128/mmbr.00080-22 36927044 PMC10304885

[4] Tan IS, Ramamurthi KS. 2014. Spore formation in Bacillus subtilis. Environ Microbiol Rep 6:212–225. doi:10.1111/1758-2229.12130 24983526 PMC4078662

[5] Khanna K, Lopez-Garrido J, Pogliano K. 2020. Shaping an endospore: architectural transformations during Bacillus subtilis sporulation. Annu Rev Microbiol 74:361–386. doi:10.1146/annurev-micro-022520-074650 32660383 PMC7610358

[6] Khanna K, Lopez-Garrido J, Zhao Z, Watanabe R, Yuan Y, Sugie J, Pogliano K, Villa E. 2019. The molecular architecture of engulfment during Bacillus subtilis sporulation. elife 8:e45257. doi:10.7554/eLife.45257 31282858 PMC6684271

[7] Broder DH, Pogliano K. 2006. Forespore engulfment mediated by a ratchet-like mechanism. Cell 126:917–928. doi:10.1016/j.cell.2006.06.053 16959571 PMC3266857

[8] Shuster B, Khemmani M, Abe K, Huang X, Nakaya Y, Maryn N, Buttar S, Gonzalez AN, Driks A, Sato T, Eichenberger P. 2019. Contributions of crust proteins to spore surface properties in Bacillus subtilis. Mol Microbiol 111:825–843. doi:10.1111/mmi.14194 30582883 PMC6417949

[9] Driks A, Eichenberger P. 2016. The spore coat. Microbiol Spectr 4. doi:10.1128/microbiolspec.TBS-0023-2016 27227299

[10] Henriques AO, Moran, Jr. CP. 2007. Structure, assembly, and function of the spore surface layers. Annu Rev Microbiol 61:555–588. doi:10.1146/annurev.micro.61.080706.093224 18035610

[11] Christie G, Setlow P. 2020. Bacillus spore germination: Knowns, unknowns and what we need to learn. Cell Signal 74:109729. doi:10.1016/j.cellsig.2020.109729 32721540

[12] Sturm A, Dworkin J. 2015. Phenotypic diversity as a mechanism to exit cellular dormancy. Curr Biol 25:2272–2277. doi:10.1016/j.cub.2015.07.018 26279233 PMC4778719

[13] Fawcett P, Eichenberger P, Losick R, Youngman P. 2000. The transcriptional profile of early to middle sporulation in Bacillus subtilis. Proc Natl Acad Sci USA 97:8063–8068. doi:10.1073/pnas.140209597 10869437 PMC16670

[14] Iwańska O, Latoch P, Kopik N, Kovalenko M, Lichocka M, Serwa R, Starosta AL. 2024. Translation in Bacillus subtilis is spatially and temporally coordinated during sporulation. Nat Commun 15:7188. doi:10.1038/s41467-024-51654-6 39169056 PMC11339384

[15] Feaga HA, Hong H-R, Prince CR, Rankin A, Buskirk AR, Dworkin J. 2023. Elongation factor P is important for sporulation initiation. J Bacteriol 205:e0037022. doi:10.1128/jb.00370-22 36651772 PMC9945569

[16] Chan H, Taib N, Gilmore MC, Mohamed AMT, Hanna K, Luhur J, Nguyen H, Hafiz E, Cava F, Gribaldo S, Rudner D, Rodrigues CDA. 2022. Genetic screens identify additional genes implicated in envelope remodeling during the engulfment stage of Bacillus subtilis sporulation. mbio 13:e01732–22. doi:10.1128/mbio.01732-22 36066101 PMC9600426

[17] Ramírez-Guadiana FH, Brogan AP, Rudner DZ. 2024. Identification and characterization of the Bacillus subtilis spore germination protein GerY. J Bacteriol 206:e0039924. doi:10.1128/jb.00399-24 39530705 PMC11656775

[18] Yu B, Kanaan J, Shames H, Wicander J, Aryal M, Li Y, Korza G, Brul S, Kramer G, Li Y, Nichols FC, Hao B, Setlow P. 2023. Identification and characterization of new proteins crucial for bacterial spore resistance and germination. Front Microbiol 14. doi:10.3389/fmicb.2023.1161604 PMC10126465 37113233

[19] Sayer CV, Barat B, Popham DL. 2019. Identification of L-Valine-initiated-germination-active genes in Bacillus subtilis using Tn-seq. PLoS One 14:e0218220. doi:10.1371/journal.pone.0218220 31199835 PMC6568419

[20] Fimlaid KA, Bond JP, Schutz KC, Putnam EE, Leung JM, Lawley TD, Shen A. 2013. Global analysis of the sporulation pathway of Clostridium difficile. PLoS Genet 9:e1003660. doi:10.1371/journal.pgen.1003660 23950727 PMC3738446

[21] Meeske AJ, Rodrigues CDA, Brady J, Lim HC, Bernhardt TG, Rudner DZ. 2016. High-throughput genetic screens identify a large and diverse collection of new sporulation genes in Bacillus subtilis. PLOS Biol 14:e1002341. doi:10.1371/journal.pbio.1002341 26735940 PMC4703394

[22] Mirouze N, Prepiak P, Dubnau D. 2011. Fluctuations in spo0A transcription control rare developmental transitions in Bacillus subtilis. PLoS Genet 7:e1002048. doi:10.1371/journal.pgen.1002048 21552330 PMC3084206

[23] Galperin MY, Yutin N, Wolf YI, Vera Alvarez R, Koonin EV. 2022. Conservation and evolution of the sporulation gene set in diverse members of the Firmicutes J Bacteriol 204:e0007922. doi:10.1128/jb.00079-22 35638784 PMC9210971

[24] Abe K, Kato H, Hasegawa Y, Yamamoto T, Nomura N, Obana N. 2022. Visualization and characterization of spore morphogenesis in Paenibacillus polymyxa ATCC39564. J Gen Appl Microbiol 68:79–86. doi:10.2323/jgam.2021.10.006 35418538

[25] Davidson P, Eutsey R, Redler B, Hiller NL, Laub MT, Durand D. 2018. Flexibility and constraint: evolutionary remodeling of the sporulation initiation pathway in Firmicutes. PLOS Genet 14:e1007470. doi:10.1371/journal.pgen.1007470 30212463 PMC6136694

[26] Hoch JA. 1993. Regulation of the phosphorelay and the initiation of sporulation in Bacillus subtilis. Annu Rev Microbiol 47:441–465. doi:10.1146/annurev.mi.47.100193.002301 8257105

[27] Burbulys D, Trach KA, Hoch JA. 1991. Initiation of sporulation in B. subtilis is controlled by a multicomponent phosphorelay. Cell 64:545–552. doi:10.1016/0092-8674(91)90238-t 1846779

[28] Sonenshein AL. 2000. Control of sporulation initiation in Bacillus subtilis. Curr Opin Microbiol 3:561–566. doi:10.1016/s1369-5274(00)00141-7 11121774

[29] Trach KA, Hoch JA. 1993. Multisensory activation of the phosphorelay initiating sporulation in Bacillus subtilis: identification and sequence of the protein kinase of the alternate pathway. Mol Microbiol 8:69–79. doi:10.1111/j.1365-2958.1993.tb01204.x 8497199

[30] Jiang M, Shao W, Perego M, Hoch JA. 2000. Multiple histidine kinases regulate entry into stationary phase and sporulation in Bacillus subtilis. Mol Microbiol 38:535–542. doi:10.1046/j.1365-2958.2000.02148.x 11069677

[31] Tzeng Y-L, Hoch JA. 1997. Molecular recognition in signal transduction: the interaction surfaces of the Spo0F response regulator with its cognate phosphorelay proteins revealed by alanine scanning mutagenesis. J Mol Biol 272:200–212. doi:10.1006/jmbi.1997.1226 9299348

[32] Kawamura F, Saito H. 1983. Isolation and mapping of a new suppressor mutation of an early sporulation gene spoOF mutation in Bacillus subtilis. Mol Gen Genet 192:330–334. doi:10.1007/BF00392171 6419021

[33] Eswaramoorthy P, Duan D, Dinh J, Dravis A, Devi SN, Fujita M. 2010. The threshold level of the sensor histidine kinase KinA governs entry into sporulation in Bacillus subtilis. J Bacteriol 192:3870–3882. doi:10.1128/JB.00466-10 20511506 PMC2916370

[34] Eswaramoorthy P, Guo T, Fujita M. 2009. In vivo domain-based functional analysis of the major sporulation sensor kinase, KinA, in Bacillus subtilis. J Bacteriol 191:5358–5368. doi:10.1128/JB.00503-09 19561131 PMC2725609

[35] Molle V, Fujita M, Jensen ST, Eichenberger P, González-Pastor JE, Liu JS, Losick R. 2003. The Spo0A regulon of Bacillus subtilis. Mol Microbiol 50:1683–1701. doi:10.1046/j.1365-2958.2003.03818.x 14651647

[36] Fujita M, Losick R. 2005. Evidence that entry into sporulation in Bacillus subtilis is governed by a gradual increase in the level and activity of the master regulator Spo0A. Genes Dev 19:2236–2244. doi:10.1101/gad.1335705 16166384 PMC1221893

[37] Steiner E, Dago AE, Young DI, Heap JT, Minton NP, Hoch JA, Young M. 2011. Multiple orphan histidine kinases interact directly with Spo0A to control the initiation of endospore formation in Clostridium acetobutylicum. Mol Microbiol 80:641–654. doi:10.1111/j.1365-2958.2011.07608.x 21401736 PMC3097173

[38] Underwood S, Guan S, Vijayasubhash V, Baines SD, Graham L, Lewis RJ, Wilcox MH, Stephenson K. 2009. Characterization of the sporulation initiation pathway of Clostridium difficile and its role in toxin production. J Bacteriol 191:7296–7305. doi:10.1128/JB.00882-09 19783633 PMC2786572

[39] Shen A, Edwards AN, Sarker MR, Paredes-Sabja D. 2019. Sporulation and germination in clostridial pathogens. Microbiol Spectr 7. doi:10.1128/microbiolspec.gpp3-0017-2018 PMC6927485 31858953

[40] Grady EN, MacDonald J, Liu L, Richman A, Yuan Z-C. 2016. Current knowledge and perspectives of Paenibacillus: a review. Microb Cell Fact 15:203. doi:10.1186/s12934-016-0603-7 27905924 PMC5134293

[41] Xie J, Shi H, Du Z, Wang T, Liu X, Chen S. 2016. Comparative genomic and functional analysis reveal conservation of plant growth promoting traits in Paenibacillus polymyxa and its closely related species. Sci Rep 6:21329. doi:10.1038/srep21329 26856413 PMC4746698

[42] Liu X, Li Q, Li Y, Guan G, Chen S. 2019. Paenibacillus strains with nitrogen fixation and multiple beneficial properties for promoting plant growth. PeerJ 7:e7445. doi:10.7717/peerj.7445 31579563 PMC6761918

[43] Cherchali A, Boukhelata N, Kaci Y, Abrous-Belbachir O, Djebbar R. 2019. Isolation and identification of a phosphate-solubilizing Paenibacillus polymyxa strain GOL 0202 from durum wheat (Triticum durum Desf.) rhizosphere and its effect on some seedlings morphophysiological parameters. Biocatalysis and Agricultural Biotechnology 19:101087. doi:10.1016/j.bcab.2019.101087

[44] Antonopoulos DF, Tjamos SE, Antoniou PP, Rafeletos P, Tjamos EC. 2008. Effect of Paenibacillus alvei, strain K165, on the germination of Verticillium dahliae microsclerotia in planta. Biol Control 46:166–170. doi:10.1016/j.biocontrol.2008.05.003

[45] Naing KW, Anees M, Kim SJ, Nam Y, Kim YC, Kim KY. 2014. Characterization of antifungal activity of Paenibacillus ehimensis KWN38 against soilborne phytopathogenic fungi belonging to various taxonomic groups. Ann Microbiol 64:55–63. doi:10.1007/s13213-013-0632-y

[46] Xu SJ, Hong SJ, Choi W, Kim BS. 2014. Antifungal activity of Paenibacillus kribbensis strain T-9 isolated from soils against several plant pathogenic fungi. Plant Pathol J 30:102–108. doi:10.5423/PPJ.OA.05.2013.0052 25288992 PMC4174836

[47] DeLeon SD, Welliver RC. 2016. Paenibacillus alvei sepsis in a neonate. Pediatr Infect Dis J 35:358. doi:10.1097/INF.0000000000001003 26866854

[48] Smith D, Bastug K, Burgoine K, Broach JR, Hammershaimb EA, Hehnly C, Morton SU, Osman M, Schiff SJ, Ericson JE. 2025. A systematic review of human Paenibacillus infections and comparison of adult and pediatric cases. Pediatr Infect Dis J 44:455–461. doi:10.1097/INF.0000000000004668 39705610 PMC11991890

[49] Paulson JN, Williams BL, Hehnly C, Mishra N, Sinnar SA, Zhang L, Ssentongo P, Mbabazi-Kabachelor E, Wijetunge DSS, von Bredow B, et al.. 2020. Paenibacillus infection with frequent viral coinfection contributes to postinfectious hydrocephalus in Ugandan infants. Sci Transl Med 12:eaba0565. doi:10.1126/scitranslmed.aba0565 32998967 PMC7774825

[50] Hehnly C, Shi A, Ssentongo P, Zhang L, Isaacs A, Morton SU, Streck N, Erdmann-Gilmore P, Tolstoy I, Townsend RR, Limbrick DD, Paulson JN, Ericson JE, Galperin MY, Schiff SJ, Broach JR. 2022. Type IV Pili Are a critical virulence factor in clinical isolates of Paenibacillus thiaminolyticus. mbio 13:e0268822. doi:10.1128/mbio.02688-22 36374038 PMC9765702

[51] Lu S, Li H, Ma C, Li X. 2025. Systemic and localized infections in humans caused by Paenibacillus: a case report and literature review. BMC Ophthalmol 25:133. doi:10.1186/s12886-025-03966-4 40087598 PMC11907811

[52] Mannava SV, DeCou J, Watkins DJ, Crumb T, Pardington E, Pennington EC. 2022. Paenibacillus wound infection in a pediatric trauma patient. J Pediatr Surg Case Rep 76:102127. doi:10.1016/j.epsc.2021.102127

[53] Vecchia ID, Fasan D, Pegoraro M, Benedetti P. 2023. Febrile sepsis: first report of human disease due to Paenibacillus silvae Access Microbiol 5:v3. doi:10.1099/acmi.0.000580.v3 PMC10323802 37424539

[54] Ebeling J, Knispel H, Hertlein G, Fünfhaus A, Genersch E. 2016. Biology of Paenibacillus larvae, a deadly pathogen of honey bee larvae. Appl Microbiol Biotechnol 100:7387–7395. doi:10.1007/s00253-016-7716-0 27394713

[55] Genersch E. 2010. American foulbrood in honeybees and its causative agent, Paenibacillus larvae. J Invertebr Pathol 103 Suppl 1:S10–9. doi:10.1016/j.jip.2009.06.015 19909971

[56] Beno SM, Cheng RA, Orsi RH, Duncan DR, Guo X, Kovac J, Carroll LM, Martin NH, Wiedmann M. 2020. Paenibacillus odorifer, the predominant Paenibacillus species isolated from milk in the United States, demonstrates genetic and phenotypic conservation of psychrotolerance but clade-associated differences in nitrogen metabolic pathways. mSphere 5:e00739. doi:10.1128/mSphere.00739-19 31969477 PMC7407005

[57] Helmond M, Nierop Groot MN, van Bokhorst-van de Veen H. 2017. Characterization of four Paenibacillus species isolated from pasteurized, chilled ready-to-eat meals. Int J Food Microbiol 252:35–41. doi:10.1016/j.ijfoodmicro.2017.04.008 28458190

[58] Guinebretiere M-H, Berge O, Normand P, Morris C, Carlin F, Nguyen-The C. 2001. Identification of bacteria in pasteurized zucchini purées stored at different temperatures and comparison with those found in other pasteurized vegetable purées. Appl Environ Microbiol 67:4520–4530. doi:10.1128/AEM.67.10.4520-4530.2001 11571151 PMC93198

[59] Elfmann C, Dumann V, Berg T, Stülke J. 2024. A new framework for SubtiWiki, the database for the model organism Bacillus subtilis. Nucleic Acids Res 53:D864–D870. doi: 10.1093/nar/gkae957 10.1093/nar/gkae957 PMC11701700 39441067

[60] Commichau FM, Pietack N, Stülke J. 2013. Essential genes in Bacillus subtilis: a re-evaluation after ten years. Mol Biosyst 9:1068–1075. doi:10.1039/c3mb25595f 23420519

[61] Fimlaid KA, Shen A. 2015. Diverse mechanisms regulate sporulation sigma factor activity in the Firmicutes. Curr Opin Microbiol 24:88–95. doi:10.1016/j.mib.2015.01.006 25646759 PMC4380625

[62] Parks DH, Chuvochina M, Rinke C, Mussig AJ, Chaumeil P-A, Hugenholtz P. 2022. GTDB: an ongoing census of bacterial and archaeal diversity through a phylogenetically consistent, rank normalized and complete genome-based taxonomy. Nucleic Acids Res 50:D785–D794. doi:10.1093/nar/gkab776 34520557 PMC8728215

[63] Li Y, Zhang D, Bo D, Peng D, Sun M, Zheng J. 2024. A taxonomic note on the order Caryophanales: description of 12 novel families and emended description of 21 families. Int J Syst Evol Microbiol 74:006539. doi:10.1099/ijsem.0.006539 39556488

[64] Schoch CL, Ciufo S, Domrachev M, Hotton CL, Kannan S, Khovanskaya R, Leipe D, Mcveigh R, O’Neill K, Robbertse B, Sharma S, Soussov V, Sullivan JP, Sun L, Turner S, Karsch-Mizrachi I. 2020. NCBI Taxonomy: a comprehensive update on curation, resources and tools. Database (Oxford) 2020:baaa062. doi:10.1093/database/baaa062 32761142 PMC7408187

[65] Park S-Y, Park S-H, Choi S-K. 2012. Characterization of sporulation histidine kinases of Paenibacillus polymyxa. Res Microbiol 163:272–278. doi:10.1016/j.resmic.2012.02.003 22391390

[66] Zapf J, Sen U Madhusudan S Hoch JA, Varughese KI. 2000. A transient interaction between two phosphorelay proteins trapped in a crystal lattice reveals the mechanism of molecular recognition and phosphotransfer in signal transduction. Structure 8:851–862. doi:10.1016/S0969-2126(00)00174-X 10997904

[67] Bach JN, Bramkamp M. 2014. Preparation of Bacillus subtilis cell lysates and membranes. Bio Protoc 4. doi:10.21769/BioProtoc.1025

[68] Evans R, O’Neill M, Pritzel A, Antropova N, Senior A, Green T, Žídek A, Bates R, Blackwell S, Yim J, Ronneberger O, Bodenstein S, Zielinski M, Bridgland A, Potapenko A, Cowie A, Tunyasuvunakool K, Jain R, Clancy E, Kohli P, Jumper J, Hassabis D. 2022. Protein complex prediction with AlphaFold-Multimer. Bioinformatics. doi:10.1101/2021.10.04.463034

[69] Karimova G, Pidoux J, Ullmann A, Ladant D. 1998. A bacterial two-hybrid system based on a reconstituted signal transduction pathway. Proc Natl Acad Sci USA 95:5752–5756. doi:10.1073/pnas.95.10.5752 9576956 PMC20451

[70] Zschiedrich CP, Keidel V, Szurmant H. 2016. Molecular mechanisms of two-component signal transduction. J Mol Biol 428:3752–3775. doi:10.1016/j.jmb.2016.08.003 27519796 PMC5023499

[71] Aseev LV, Koledinskaya LS, Boni IV. 2016. Regulation of ribosomal protein operons rplM-rpsI, rpmB-rpmG, and rplU-rpmA at the transcriptional and translational levels. J Bacteriol 198:2494–2502. doi:10.1128/JB.00187-16 27381917 PMC4999927

[72] Nanamiya H, Akanuma G, Natori Y, Murayama R, Kosono S, Kudo T, Kobayashi K, Ogasawara N, Park S-M, Ochi K, Kawamura F. 2004. Zinc is a key factor in controlling alternation of two types of L31 protein in the Bacillus subtilis ribosome. Mol Microbiol 52:273–283. doi:10.1111/j.1365-2958.2003.03972.x 15049826

[73] Trach K, Hoch JA. 1989. The Bacillus subtilis spo0B stage 0 sporulation operon encodes an essential GTP-binding protein. J Bacteriol 171:1362–1371. doi:10.1128/jb.171.3.1362-1371.1989 2537815 PMC209754

[74] Vidwans SJ, Ireton K, Grossman AD. 1995. Possible role for the essential GTP-binding protein Obg in regulating the initiation of sporulation in Bacillus subtilis. J Bacteriol 177:3308–3311. doi:10.1128/jb.177.11.3308-3311.1995 7768831 PMC177024

[75] Kok J, Trach KA, Hoch JA. 1994. Effects on Bacillus subtilis of a conditional lethal mutation in the essential GTP-binding protein Obg. J Bacteriol 176:7155–7160. doi:10.1128/jb.176.23.7155-7160.1994 7961486 PMC197102

[76] Stephenson K, Hoch JA. 2002. Evolution of signalling in the sporulation phosphorelay. Mol Microbiol 46:297–304. doi:10.1046/j.1365-2958.2002.03186.x 12406209

[77] Mattoo AR, Saif Zaman M, Dubey GP, Arora A, Narayan A, Jailkhani N, Rathore K, Maiti S, Singh Y. 2008. Spo0B of Bacillus anthracis - a protein with pleiotropic functions. FEBS J 275:739–752. doi:10.1111/j.1742-4658.2007.06240.x 18190531

[78] Coleman GA, Davín AA, Mahendrarajah TA, Szánthó LL, Spang A, Hugenholtz P, Szöllősi GJ, Williams TA. 2021. A rooted phylogeny resolves early bacterial evolution. Science 372. doi:10.1126/science.abe0511 33958449

[79] Capra EJ, Laub MT. 2012. Evolution of two-component signal transduction systems. Annu Rev Microbiol 66:325–347. doi:10.1146/annurev-micro-092611-150039 22746333 PMC4097194

[80] da Costa RA, Andrade IEPC, de Araújo TF, Fulgêncio DLA, Mendonça ML, Rocha GIY, Dos Santos RM, Barreto CC. 2024. Correlation between sporogenesis and lipopeptide production in Paenibacillus elgii. Lett Appl Microbiol 77:ovae079. doi:10.1093/lambio/ovae079 39191532

[81] Mercl F, Tejnecký V, Ságová-Marečková M, Dietel K, Kopecký J, Břendová K, Kulhánek M, Košnář Z, Száková J, Tlustoš P. 2018. Co-application of wood ash and Paenibacillus mucilaginosus to soil: the effect on maize nutritional status, root exudation and composition of soil solution. Plant Soil 428:105–122. doi:10.1007/s11104-018-3664-z

[82] Aw Y-K, Ong K-S, Lee L-H, Cheow Y-L, Yule CM, Lee S-M. 2016. Newly isolated Paenibacillus tyrfis sp. nov., from Malaysian tropical peat swamp soil with broad spectrum antimicrobial activity. Front Microbiol 7:219. doi:10.3389/fmicb.2016.00219 26973605 PMC4771734

[83] LeDeaux JR, Yu N, Grossman AD. 1995. Different roles for KinA, KinB, and KinC in the initiation of sporulation in Bacillus subtilis. J Bacteriol 177:861–863. doi:10.1128/jb.177.3.861-863.1995 7836330 PMC176674

[84] Varughese KI Madhusudan S Zhou XZ, Whiteley JM, Hoch JA. 1998. Formation of a novel four-helix bundle and molecular recognition sites by dimerization of a response regulator phosphotransferase. Mol Cell 2:485–493. doi:10.1016/S1097-2765(00)80148-3 9809070

[85] Hoch JA, Varughese KI. 2001. Keeping signals straight in phosphorelay signal transduction. J Bacteriol 183:4941–4949. doi:10.1128/JB.183.17.4941-4949.2001 11489844 PMC95367

[86] Gaidenko TA, Kim T-J, Price CW. 2002. The PrpC serine-threonine phosphatase and PrkC kinase have opposing physiological roles in stationary-phase Bacillus subtilis cells. J Bacteriol 184:6109–6114. doi:10.1128/JB.184.22.6109-6114.2002 12399479 PMC151969

[87] Koo B-M, Kritikos G, Farelli JD, Todor H, Tong K, Kimsey H, Wapinski I, Galardini M, Cabal A, Peters JM, Hachmann A-B, Rudner DZ, Allen KN, Typas A, Gross CA. 2017. Construction and analysis of two genome-scale deletion libraries for Bacillus subtilis. Cell Syst 4:291–305. doi:10.1016/j.cels.2016.12.013 28189581 PMC5400513

[88] Ash C, Priest FG, Collins MD. 1993. Molecular identification of rRNA group 3 bacilli (Ash, Farrow, Wallbanks and Collins) using a PCR probe test. Antonie Van Leeuwenhoek 64:253–260. doi:10.1007/BF00873085 8085788

[89] Li W, O’Neill KR, Haft DH, DiCuccio M, Chetvernin V, Badretdin A, Coulouris G, Chitsaz F, Derbyshire MK, Durkin AS, Gonzales NR, Gwadz M, Lanczycki CJ, Song JS, Thanki N, Wang J, Yamashita RA, Yang M, Zheng C, Marchler-Bauer A, Thibaud-Nissen F. 2021. RefSeq: expanding the prokaryotic genome annotation pipeline reach with protein family model curation. Nucleic Acids Res 49:D1020–D1028. doi:10.1093/nar/gkaa1105 33270901 PMC7779008

[90] Parks DH, Imelfort M, Skennerton CT, Hugenholtz P, Tyson GW. 2015. CheckM: assessing the quality of microbial genomes recovered from isolates, single cells, and metagenomes. Genome Res 25:1043–1055. doi:10.1101/gr.186072.114 25977477 PMC4484387

[91] Lee MD. 2019. GToTree: a user-friendly workflow for phylogenomics. Bioinformatics 35:4162–4164. doi:10.1093/bioinformatics/btz188 30865266 PMC6792077

[92] Yu G, Smith DK, Zhu H, Guan Y, Lam T-Y. 2017. GGTREE:an R package for visualization and annotation of phylogenetic trees with their covariates and other associated data. Methods Ecol Evol 8:28–36. doi:10.1111/2041-210X.12628

[93] Schliep KP. 2011. Phangorn: phylogenetic analysis in R. Bioinformatics 27:592–593. doi:10.1093/bioinformatics/btq706 21169378 PMC3035803

[94] Altschul SF, Gish W, Miller W, Myers EW, Lipman DJ. 1990. Basic local alignment search tool. J Mol Biol 215:403–410. doi:10.1016/S0022-2836(05)80360-2 2231712

[95] Finn RD, Clements J, Eddy SR. 2011. HMMER web server: interactive sequence similarity searching. Nucleic Acids Res 39:W29–37. doi:10.1093/nar/gkr367 21593126 PMC3125773

[96] Hallgren J, Tsirigos KD, Pedersen MD, Almagro Armenteros JJ, Marcatili P, Nielsen H, Krogh A, Winther O. 2022. DeepTMHMM predicts alpha and beta transmembrane proteins using deep neural networks. Bioinformatics. doi:10.1101/2022.04.08.487609

[97] Gasteiger E, Hoogland C, Gattiker A, Duvaud S, Wilkins MR, Appel RD, Bairoch A. 2005. Protein Identification and Analysis Tools on the ExPASy Server, p 571–607. In Walker JM (ed), The Proteomics Protocols Handbook. Humana Press, Totowa, NJ.

[98] Abramson J, Adler J, Dunger J, Evans R, Green T, Pritzel A, Ronneberger O, Willmore L, Ballard AJ, Bambrick J, et al.. 2024. Accurate structure prediction of biomolecular interactions with AlphaFold 3. Nature 630:493–500. doi:10.1038/s41586-024-07487-w 38718835 PMC11168924

[99] Meng EC, Goddard TD, Pettersen EF, Couch GS, Pearson ZJ, Morris JH, Ferrin TE. 2023. UCSF ChimeraX: Tools for structure building and analysis. Protein Sci 32:e4792. doi:10.1002/pro.4792 37774136 PMC10588335

[100] Sayers EW, Beck J, Bolton EE, Brister JR, Chan J, Connor R, Feldgarden M, Fine AM, Funk K, Hoffman J, et al.. 2025. Database resources of the National Center for biotechnology information in 2025. Nucleic Acids Res 53:D20–D29. doi:10.1093/nar/gkae979 39526373 PMC11701734

[101] Sievers F, Higgins DG. 2018. Clustal Omega for making accurate alignments of many protein sequences. Protein Sci 27:135–145. doi:10.1002/pro.3290 28884485 PMC5734385

[102] Shimoyama Y. 2022. pyMSAviz: MSA visualization python package for sequence analysis. https://github.com/moshi4/pyMSAviz.

[103] Grant BJ, Rodrigues APC, ElSawy KM, McCammon JA, Caves LSD. 2006. Bio3d: an R package for the comparative analysis of protein structures. Bioinformatics 22:2695–2696. doi:10.1093/bioinformatics/btl461 16940322

[104] Katoh K, Standley DM. 2013. MAFFT multiple sequence alignment software version 7: improvements in performance and usability. Mol Biol Evol 30:772–780. doi:10.1093/molbev/mst010 23329690 PMC3603318

[105] Schneider CA, Rasband WS, Eliceiri KW. 2012. NIH Image to ImageJ: 25 years of image analysis. Nat Methods 9:671–675. doi:10.1038/nmeth.2089 22930834 PMC5554542

