# Supplementary material for: Paenibacillus encodes a membrane-localized Spo0B
Source: J Bacteriol. 2025 Dec 29;208(1):e00367-25. doi: 10.1128/jb.00367-25 (PMC12826061; doi:10.1128/jb.00367-25)
Supplement: Supplemental table legends — Legends for Tables S1 to S5. [file jb.00367-25-s0002.docx]

**Supplemental table legends**

**Table S1.** *Paenibacillus* spp. genome accession numbers.

**Table S2.** Conservation of *B. subtilis* sporulation genes in *Paenibacillus*.

**Table S3.** Essential sporulation genes in *B. subtilis* that are absent from the *Paenibacillus* core sporulation genes.

**Table S4.** Bacilli genome accession numbers and Spo0B variant data.

**Table S5.** Protein accession numbers for Spo0B HMMER searches, AlphaFold3 models in Figure 3A and 5A, and sequence alignments in Figures 3B and 7A, and genome accessions for synteny in Figure 6A.
